# Dermatology

**Published:** 1904-10-15

**Authors:** 


### Progress in Medicine and Surgery.

### DERMATOLOGY.

**Seborrhœa.**—So much has been written upon seborrhœa that authors who write upon this subject preface their remarks with apologies. The difficulty mainly appears to lie in the definition. The term may be equally applied to "a greasy affection of certain areas, accompanied by dilatation and plugging of the pilo-sebaceous follicles"; the seborrhœa corporis of Duhring; seborrhœa capitis; to psoriatic figured eruptions when the scales are greasy and yellowish; and even the seborrhœa sicca of Hebra (Brooke). Until the condition has been more definitely defined, much fruitless discussion will ensue. Neither Brooke [1] nor Whitfield [2] can accept the microbacillus of Sabouraud, swarming though it may be in the oily secretion, as the actual cause of the seborrhœa. Brooke considers it as a steatophilic saprophyte. Audry, he says, rejects the microbic causation of the seborrhœa of Sabouraud, and regards it as no more than a congenital abnormality. While coinciding in the main, Brooke takes exception to the term "abnormality," preferring to call it a "stage" in the evolution or degeneration of the human organism, in which the glands are adjusting themselves to a changing environment. Brooke's own view on the subject is more as follows:—The main features of seborrhœa are Sabouraud's cocoons, horny plugs in the pilo-sebaceous infundibula, associated with abnormal greasiness. The exact clinical picture presented depends upon external causes, such as pustulation and inflammation. On the face there is quite a different picture from on the elbow, even though the condition of follicular papules and plugging may be present in both, the difference depending upon the variety in the number of the follicles. Anywhere and at any stage "the eruption may become acutely eczematous, and spread as a papular rash without particular form." Brooke has watched on several occasions the development of psoriasis from circinate seborrhœa corporis eruptions, as also the converse, when in a patient with typical psoriasis an acute development of Unna's "eczema seborrhoicum" has occurred. He sums up his observations in the following words: "There is an affection of the epidermis characterised by abnormal greasiness and the formation of horny plugs in the pilo-sebaceous infundibula. There is a series of pityriasic eruptions beginning with a simple patchy or circinate pityriasis and ending by a series of unbroken steps with psoriasis. These certainly have the aspect of microbic affections and may be "psoriasides" in a wide sense; they may become eczematous, especially when developed in conjunction with the seborrhoic state, but are not eczema; they may be influenced in their aspect and evolution by the seborrhoic condition, but are not seborrhoides, unless that term be confined strictly to those cases in which they are combined with the seborrhoic conditions to form a seborrhoic dermatitis. Whitfield [3] states that there is now a certain concensus of opinion that the organism causing the eruption of seborrhœa corporis, or seborrhoic eczema, is a staphylococcus which does not liquefy gelatine, but produces a smell of butyric acid in culture. The actual lesions may be therefore due to the decomposition of the secretion rather than to actual infection, or the organism may increase in virulence under suitable conditions of skin.

**Megalerythema Epidemicum or Erythema Infectiosum.**—This disease, as described by Plachte,[4] is characterised by rounded patches of erythema, raised and bright red in colour, with edges not sharply defined, without pain or itching. The spots tend to fade in the centre in two days, the raised centre becoming depressed and the red colour giving place to a brownish-red tinge. As the centre fades the patches become annular, the width of the ring growing gradually smaller. The eruption in the case recorded by Plachte occurred on the face of a girl, aged three, in patches the size of a finger-nail; but after fading from the face the eruption appeared upon the limbs, attaining here a much larger size (= five shillings), and showing a marked tendency to become confluent. The trunk was affected last. Over the sternum there was slight desquamation, but otherwise none. A few relapses occurred about the body and recovery was complete in 8 or 9 days. The mucous membranes were not involved, and no pyrexia appeared. There were slight pains in the limbs, otherwise no disturbance of general health. The other members of the family consisted of 5 adults and 3 children. The adults were not affected while the children all suffered. The oldest, æt. 7, first had it, 8 days later her sister, æt. 3, and 6 days later the twin sister had it. In the two earlier cases the body was not affected. This form of infectious erythema is not unknown, 30 cases having been described by Tchamer in 1886. Thirty of these were in adult women during an epidemic of rötheln in Graz. In 1889-90 17 more cases were observed in Graz during an epidemic of morbilli. It was then considered a local form of rötheln, but Sticker in 1899 published 45 cases from Giessen under the name of erythema infectiosum, as a new infectious disease in children. This type corresponds in the main with

Plachte's present case, though occasionally slight itching, initial rise of temperature, sore throat, and confluence of erythema to form uniform scarlet redness, and decided pains in the limbs were noted. All the cases occurred towards the end of April and beginning of May. Cases of megalerythema have again occurred at Graz during an outbreak of rötheln. A. Schmidt has shown that neither rötheln nor morbilii protect against the disease, and proclaims it a separate entity. Tripke, of Coblenz, has lately described an epidemic of 60 cases, under the name of erythema infectiosum febrile. Here, however, there was an initial rise of temperature to 104° F. The latest outbreak was described by Feilchenfeld under the name of erythema simplex marginatum: six cases were here affected living close to each other. The points of diagnosis between this disease and erythema multiforme, which it most closely resembles are:—(1) E. multiforme affects the whole body simultaneously, whereas megalerythema affects certain positions in a definite order; (2) E. multiforme does not occur as an epidemic; (3) the spots of megalerythema are circular, and do not present the variety of erythema multiforme; (4) duration of E. multiforme is two to six weeks, of megalerythema nine days. The prognosis is good; no fatal results have been recorded.

**Blastomycetic Dermatitis.**—Gilchrist[5] claims that unless the disease is general it can be cured by the administration of pot. iod. internally, and the local application of *x*-rays until a mild "burn" is produced. Four cases of general infection have been recorded, the blastomyces having in all probability obtained entrance through the lungs. In one of Gilchrist's cases there was a relapse, owing to the treatment having been discontinued on the disappearance of the symptoms. It seems, then, that the treatment must be maintained long after apparent cure.

Lutati[6] has experimented upon the action of pure preparations of tar, oil of cade, gallacetophenol, anthrarobin, and ichthyol by applying them to the combs of cocks. Tar caused thickening of the horny layer and œdema of the cells of the granular layer. The cells' outlines diappear, the nuclei stain badly, and there are masses of nuclear *débris*. The deeper cells are swollen, with clear nuclei, showing karyokinesis. Amorphous masses of pigment were found to be due to penetration of the drug into the deeper cutis, showing that the action is a penetrative one. Henri Grenet draws attention to certain nervous reactions recorded in cases of exanthematous purpura.[7] (1) Symmetrical purpura which alternated with an attack of zona; (2) purpura, petechial rash died away and was followed by herpes of head and eyes, lumbar puncture revealed distinct, lymphocytosis and a slight cloud of albumin in the cerebrospinal fluid; (3) recurrent purpura in a tubercular patient, lumbar puncture showed presence of numerous lymphocytes and mononuclear leucocytes; (4) recurrent purpura in tubercular patient, three attacks of purpura each time on getting out of bed; during an attack cerebrospinal fluid showed a thick cloud of albumin; (5) Icterus two years ago, intermittent fever two months ago, eight days ago pain in legs followed by erythema leaving petechiæ; cerebrospinal fluid showed lymphocytosis and a fair cloud of albumin. Improvement was rapid and later the lymphocytes had almost disappeared and there was no albumin; (6) child, three and-a-half, son of a hæmophilic mother, six brothers and sisters not hæmophilic. Had epistaxis on admission, with petechial eruption with large hæmorrhages on the back, etc. Lumbar puncture revealed clear lymphocytosis and a thick cloud of albumin. The patient improved, but a second puncture showed a marked lymphocytosis and a large quantity of albumin still. Four days later the patient had an attack of herpes under the nose, which spread irregularly over the face, but without pain. Two days after the vesicles were dried a third puncture showed only a few lymphocytes and a very faint cloud of albumin. These observations are contrary to the observations of M. Leredde and others, who hold that the changes in the vessels due to the toxins circulating in the blood, are sufficient to explain the purpura The author considers that the nervous system plays a large part in every case of purpura.

**Antistaphylococcic Serum.**—A. E. Wright and Captain Douglas[8] endeavour to cure the subjects of staphylococcic and tubercular lesions, by inoculations with the dead cultures of the causative bacteria, at the same time they keep a strict observation upon the phagocytic power of the blood-cells, and estimate the dosage by these means. They from start to finish endeavour to calculate exactly the number of bacteria administered in each dose, and by making frequent blood-counts and agglutination tests before and after inoculation, are able to depict curves indicating the resistance of the body to the toxin as compared to the patients' previous state, and the normal as exemplified by a healthy individual. The cure of disease can be produced by one of two ways: the destruction *in situ* of the disease itself from externally, or the raising of the resistance of the body to such a pitch that the soil becomes unsuitable for the bacteria. The object of these inoculations is to endeavour to call out the latent resistance in the individual, a resistance which is diminished by the local ravages of the bacteria. Wright has drawn off small amounts of blood and pus from patients with tubercular or staphylococcic disease and has centrifugalised them. He has then measured the amount of protective substance in the clear fluid. While there is scarcely any protective substance in the clear fluid of the pus, there is much more in the blood. There may be too little protective substance in the blood of a patient, or there may be too limited access of the charged lymph to the infected spot. If the normal amount of protective substance in the tissues be represented as 1, it is found that the quantity in the blood of a man, the subject of staphylococcic invasion will be ·3 or ·4, etc. After the injection of the "vaccine" there is a negative phase in which there is a temporary diminution of the protective substance. This is the period prior to stimulation, in which the protective substance available in the blood is all used up. At this time the patient is liable to an outbreak of acne pustules, etc., and an aggravation of his condition. Great care must therefore be exercised to make this negative phase a short one. It is followed by an increased phagocytic power, which however does not remain uniform, though rarely falling to the same level again as before. Further inoculations gradually raise the level of the "positive" phase line until

ARMY MEDICAL LIBRARY WASHINGTON, D. C. U. S. A.

in a satisfactory case, the resisting power of the blood is as high as in the normal individual. The patient at this point is found to have lost his lesions, though fresh developments may occur later requiring further inoculations. Overplying the system with vaccine ruins the result, the whole resistance being lost, which necessitates the inoculations being performed over again. As far as technique is concerned a culture of staphylococci is made (possibly, if refractory, from the case in point itself), the cocci are killed by keeping the culture at a temperature of 60° for sufficient time, which does not alter the chemical bodies; $\frac{1}{2}$ per cent. Ac. Carbol. is added to prevent contamination. The bacteria are counted by mixing equal volumes of blood and a sterilised bacterial culture in suspension. The relative numbers of bacteria and red cells is easily estimated. With tubercle bacilli the matter is not so simple. When typhoid bacteria are injected in this way one obtains an increase of 2,000 times in the bactericidal power of the blood. The blood gains nothing, however, in bactericidal power in staphylococcic injections. But a substance is produced which paralyses the bacteria and makes them subject to phagocytosis. Such substances he proposes to call "Opsonins." They are developed in the serum, the white corpuscles not being affected. In speaking of this treatment Wright[9] says:—"It is, I think, satisfactorily established that chronic staphylococcus invasions, and in particular furunculosis, sycosis, and acne, can be treated in a very effective manner by inoculations of a staphylococcus vaccine."

[1] Brit. Jour. Dernat., June 1904. [2] Lancet, March 5. [3] Practitioner, March 1904. [4] Berlin Klin. Woch., Feb. 29, p. 223; Med. Review, April. [5] Med. Review, May. [6] Treatment, July. [7] Gazette des Hôpitaux, Thursday, August 4th, 1904. [8] Brit. Jour. Dermat., August 1904. [9] Brit. Med. Jour., May 7.

